# Aging and pathological aging signatures of the brain: through the focusing lens of SIRT6

**DOI:** 10.18632/aging.202755

**Published:** 2021-03-09

**Authors:** Daniel Stein, Amir Mizrahi, Anastasia Golova, Adam Saretzky, Alfredo Garcia Venzor, Zeev Slobodnik, Shai Kaluski, Monica Einav, Ekaterina Khrameeva, Debra Toiber

**Affiliations:** 1 Department of Life Sciences, Ben-Gurion University of the Negev, Beer Sheva 84105, Israel; 2 The Zlotowski Center for Neuroscience, Ben-Gurion University of the Negev, Beer Sheva 84105, Israel; 3 Center of Life Sciences, Skolkovo Institute of Science and Technology, Moscow 121205, Russia

**Keywords:** SIRT6, YY1, aging, neurodegeneration, transcription regulation

## Abstract

Brain-specific SIRT6-KO mice present increased DNA damage, learning impairments, and neurodegenerative phenotypes, placing SIRT6 as a key protein in preventing neurodegeneration. In the aging brain, SIRT6 levels/activity decline, which is accentuated in Alzheimer’s patients. To understand SIRT6 roles in transcript pattern changes, we analyzed transcriptomes of young WT, old WT and young SIRT6-KO mice brains, and found changes in gene expression related to healthy and pathological aging. In addition, we traced these differences in human and mouse samples of Alzheimer’s and Parkinson’s diseases, healthy aging and calorie restriction (CR). Our results define four gene expression categories that change with age in a pathological or non-pathological manner, which are either reversed or not by CR. We found that each of these gene expression categories is associated with specific transcription factors, thus serving as potential candidates for their category-specific regulation. One of these candidates is YY1, which we found to act together with SIRT6 regulating specific processes. We thus argue that SIRT6 has a pivotal role in preventing age-related transcriptional changes in brains. Therefore, reduced SIRT6 activity may drive pathological age-related gene expression signatures in the brain.

## INTRODUCTION

During aging, there is an increase in the incidence of several age-related diseases including neurodegeneration, in which aging itself is the main risk factor [1]. Technological advances over the last centuries have drastically changed the environment in which humans evolved. As a result, the mean life expectancy increased from 40-45 years before the modern era to 78.8 nowadays [2], mostly due to decreased rates of infant mortality, improved medical care, and favorable environmental conditions [3, 4]. Suddenly (in evolutionary terms), aging people have to deal with new health threats and the maintenance of organismal function for longer times, as more persons survive much longer than before. Among the few successful treatments to delay aging, Calorie Restriction (CR) has been proven successful in extending lifespan and alleviate some of the detrimental effects of aging, such as cardiovascular disease, insulin resistance, and increased oxidative damage [5]. The effects of CR are documented in various model organisms including worms, flies, mice and the primate rhesus monkey [6], suggesting that these effects are translatable into humans. Nevertheless, the underlying mechanism is still under debate.

To understand the molecular mechanism of aging, several genes have been studied – specifically, the Sirtuin deacylase and ADP-ribosylase gene family, which are highly conserved proteins with important roles in preventing age-related diseases [7–9]. For example, Sirtuins play major roles in metabolism and have been implicated in the improvement of CR response in aging. In addition, several age-related diseases have been associated with the lack of Sirtuins. Particularly, the absence of SIRT6 results in neurodegeneration [10], heart disease [11], inflammation [12], diabetes [13], and more. SIRT6 knockout mice showed accelerated aging and premature death by three weeks of age, accompanied by metabolic defects, genomic instability, and a progeroid-like phenotype [14]. Furthermore, male mice overexpressing SIRT6 display an increased lifespan [15], suggesting that SIRT6 has critical roles in the regulation of age-related pathologies. We recently showed that SIRT6 is a DNA damage sensor that can recognize Double Strand Breaks (DSB) and initiate the DNA damage response for both homologous recombination and non-homologous end joining [16, 17]. Moreover, SIRT6 has several roles in DNA repair [14, 18, 19], and its catalytic activity in DSB repair is directly involved in the evolution of longevity in long-lived mammals [20].

Since one of the driving forces of aging is the gradual accumulation of DNA damage, we speculated that brain-specific SIRT6-deficient mice could be used as a model for sporadic neurodegeneration. Indeed, this animal model presents accelerated DNA damage accumulation, learning and memory impairments, increased neuronal cell death, and the appearance of hyper-phosphorylated Tau and hyper-acetylated Tau [10, 21]. Similarly, the two most prevalent age-related neurodegenerative diseases – Parkinson's disease (PD) and Alzheimer's disease (AD) – show increased accumulation of DNA damage, epigenetic changes that lead to altered transcriptional deregulation, and some form of pathological Tau [22–25]. Altogether, these data point out SIRT6 importance in healthy brain aging.

Therefore, we used SIRT6 as a focusing lens to understand the changes in gene expression that could lead to pathological brain aging. Overall, we found that changes due to the lack of SIRT6 are commonly seen in aging and age-related neurodegenerative diseases. We classified the results into four main gene expression categories that change with age, either in a pathological or a non-pathological age-related manner, and groups of genes whose age-related expression changes are either or not reversed by CR. Our results underline a specific pathological signature that could be further used to predict the risk for developing a neurodegenerative disease.

## RESULTS

### Gene expression changes in SIRT6-KO mouse brains

**Figure 1:**
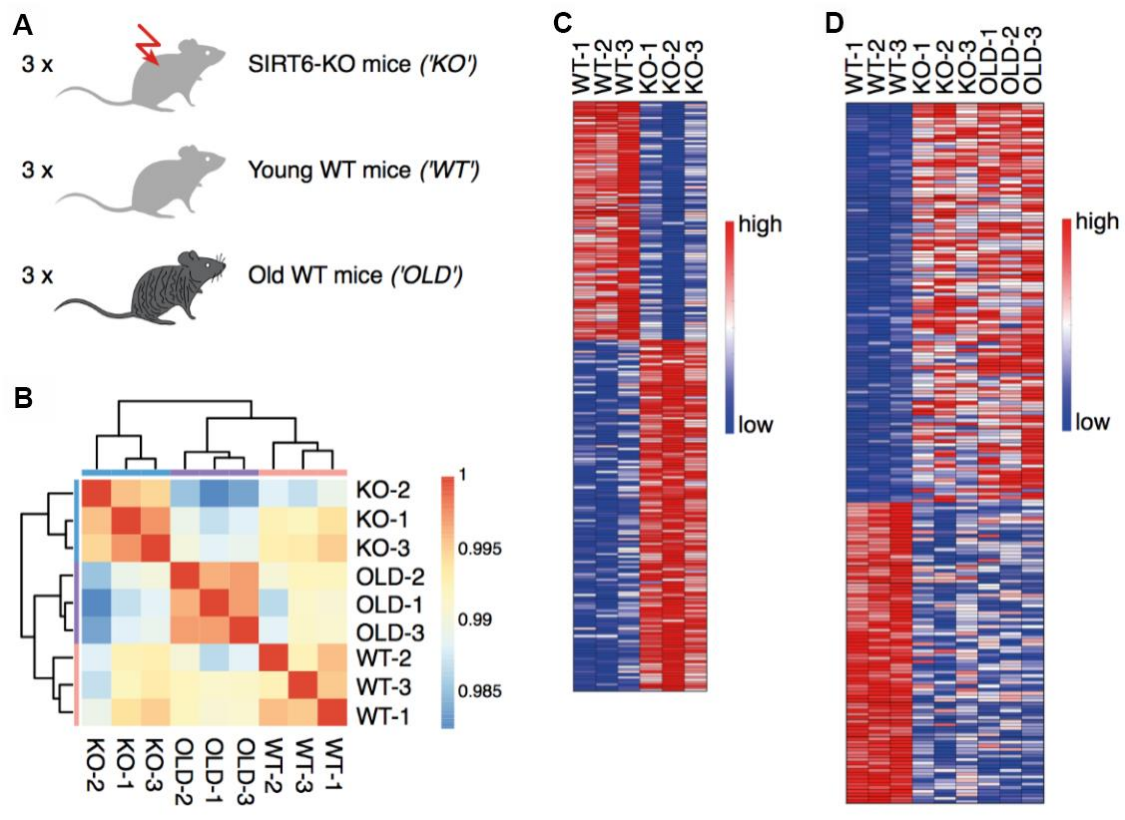
**SIRT6 deletion affects gene expression in the brain.** (**A**) Experimental design: RNA was isolated from the brains of three SIRT6-KO mice, three age-matched WT mice (21 days), and three old WT mice (22-26 months). (**B**) Hierarchical clustering of samples. Colors represent Pearson correlation coefficients. (**C**) Expression levels of genes identified as upregulated or downregulated in a comparison between SIRT6-KO and young WT mice. Red color corresponds to increased expression, while blue color corresponds to decreased expression. (**D**) Expression levels of genes identified as upregulated or downregulated in a pool of SIRT6-KO and old WT mice compared to young WT mice. Colors are as in (**C**).

To characterize the aging signature of the brain, we measured gene expression in three young WT mice (21 days), three SIRT6-KO mice of the same age, and three old WT mice (22-26 month) (submit dataset number, Figure 1A). The hierarchical clustering of samples demonstrated a high consistency between the biological replicates (Figure 1B). To separate pathological expression patterns from non-pathological ones, two comparisons were made. First, we compared SIRT6-KO to young WT mouse brain samples and identified 137 upregulated genes and 93 downregulated genes (Figure 1C). Alternative cutoffs resulted in 1811 upregulated genes and 1969 downregulated genes (see Methods for all cutoffs). Interestingly, some genes showed similar trends when we compared a pool of SIRT6-KO and Old mice with WT mice (Figure 1D). Altered regulation, in this case, can be attributed to either pathological or non-pathological aspects of SIRT6 loss of function.

To assess differences that are exclusively due to the absence of SIRT6, we compared Geneset Enrichment Analysis (GSEA) gene categories that are significantly changed in SIRT6KO in relation to both WT and aged mice groups. Both these comparisons only show the downregulation of significant categories, which we decided to further inspect (Supplementary Figure 1A, 1B). In the first place, we observe parallel changes between SIRT6KO, HDAC1 and HDAC2 knockout experiments [26], probably due to their common deacetylase activity. Additionally, many markers of oligodendrocytes and astrocytes are downregulated in SIRT6KO mice brains (Supplementary Figure 1C), pointing out the reduced amount of these cells or their activity. This reduction unearths the misregulation of neuron support infrastructures such as myelination and nutrient supply. Thus, not only the neuronal system itself is affected by SIRT6 deficiency, but its support system as well.

### Clustering of GSEA categories

**Figure 2:**
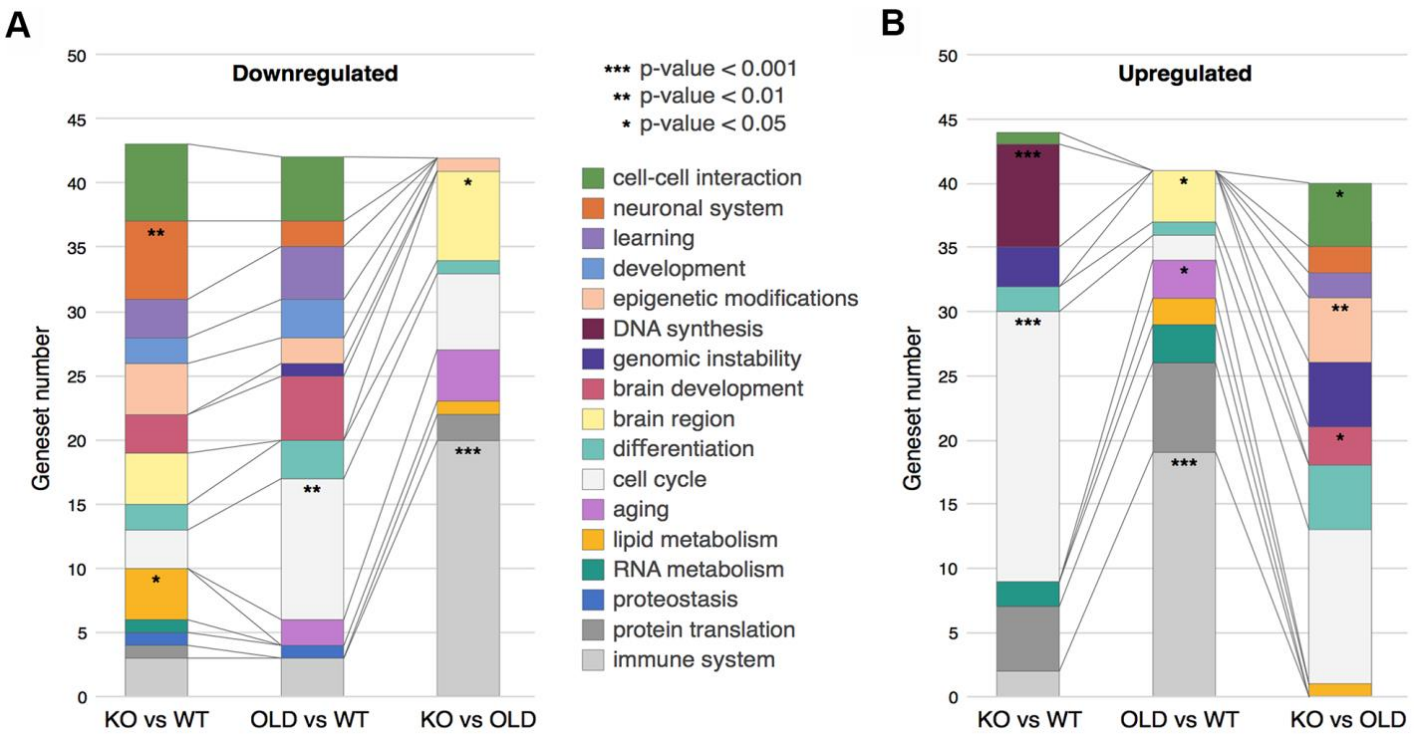
**Clustering of GSEA categories.** (**A**) Downregulated clusters of GSEA categories in the different comparisons. (**B**) Upregulated clusters of GSEA categories in the different comparisons. In both panels, colors correspond to clusters of GSEA categories, while stars represent hypergeometric test p-values: * - p-value < 0.05, ** - p-value < 0.01, *** - p-value < 0.001.

To better understand the changes occurring in our samples, we sorted the GSEA Genesets into Geneset Clusters (Figure 2A, 2B and Supplementary Table 1). In brief, we categorized the top 50 Genesets from the GSEA (per comparison) for both upregulated and downregulated genes, regardless of their p-values. Then, we clustered together these Genesets from the same biological processes such as cell cycle, cancer, immune response, etc., and identified the most enriched clusters (see Methods). For example, if interferon response and *NFKB* were in the top 50 categories, we clustered them together as immune response (Supplementary Table 2). This procedure allowed us to determine the main changes occurring in SIRT6-KO and aging brains in a systemic manner. We noticed that the immune response cluster is drastically upregulated in old mice compared to both WT and SIRT6-KO mice, possibly due to the accumulated inflammation in aging, while SIRT6-KO mice showed only a slight enrichment compared to WT, denoting some similarity with aging, although not to the full extent as old mice.

The two most upregulated clusters in SIRT6-KO mouse brains are *cell cycle* and *DNA synthesis*. Since healthy neurons do not divide, these clusters might suggest cell cycle re-entry and hyperploidy. Neurons – when stressed by DNA damage, Amyloid-beta or Tau overexpression –can initiate DNA synthesis and lead to hyperploidy (DNA synthesis without cell division) [27–29], and although we don’t fully prove this hypothesis, it is interesting to speculate why KO present increased synthesis. Importantly, these phenomena are characteristic of pathological aging brains and are highly increased in AD patients. Interestingly, this cluster was not present in old mice, emphasizing SIRT6-KO as a model for pathological aging and neurodegeneration.

Aging is characterized with an increase in genomic instability. DNA damage is considered one of the hallmarks of aging and a driving force in neurodegeneration [32]. DNA damage accumulation is increased even more in many age-related brain pathologies [33]. Not surprisingly, then, our results show that the genomic instability cluster is upregulated in SIRT6-KO when compared to WT mice, and even when compared to old mice. This observation highlights the cellular requirement to increase the expression of DNA repair genes to deal with the genomic instability due to SIRT6 absence, again pointing out DNA damage in the pathological nature of SIRT6 deficiency.

Interestingly, the top two categories downregulated in SIRT6-KO mice brains are brain-related (cell-cell interaction and neuronal system), and the learning cluster was also affected by SIRT6-KO – which indicates the cognitive decline of these mice [10].

It is well known that the protein quality control machinery deteriorates in aging, and to an even greater extent in many brain pathologies. Here, we observe that protein translation cluster is considerably elevated in SIRT6-KO and old mice brains, while the proteostasis cluster is downregulated. Our results imply that higher translation levels accompanied by lower proteostasis maintenance capacity are quite deleterious to the cell. In the long term, this could lead to proteostasis loss and, eventually, to cellular death.

We believe that our cluster analysis helps to visualize the most important changes in SIRT6-KO and aging mice, as reflected by the dynamics between the different gene expression groups. Overall, the extreme genomic instability, proteostasis loss, and misbalanced metabolism, which are evident in SIRT6-KO mice, support SIRT6 as a driver of pathological aging and neurodegeneration.

### Pathological aspects of SIRT6 loss of function

**Figure 3:**
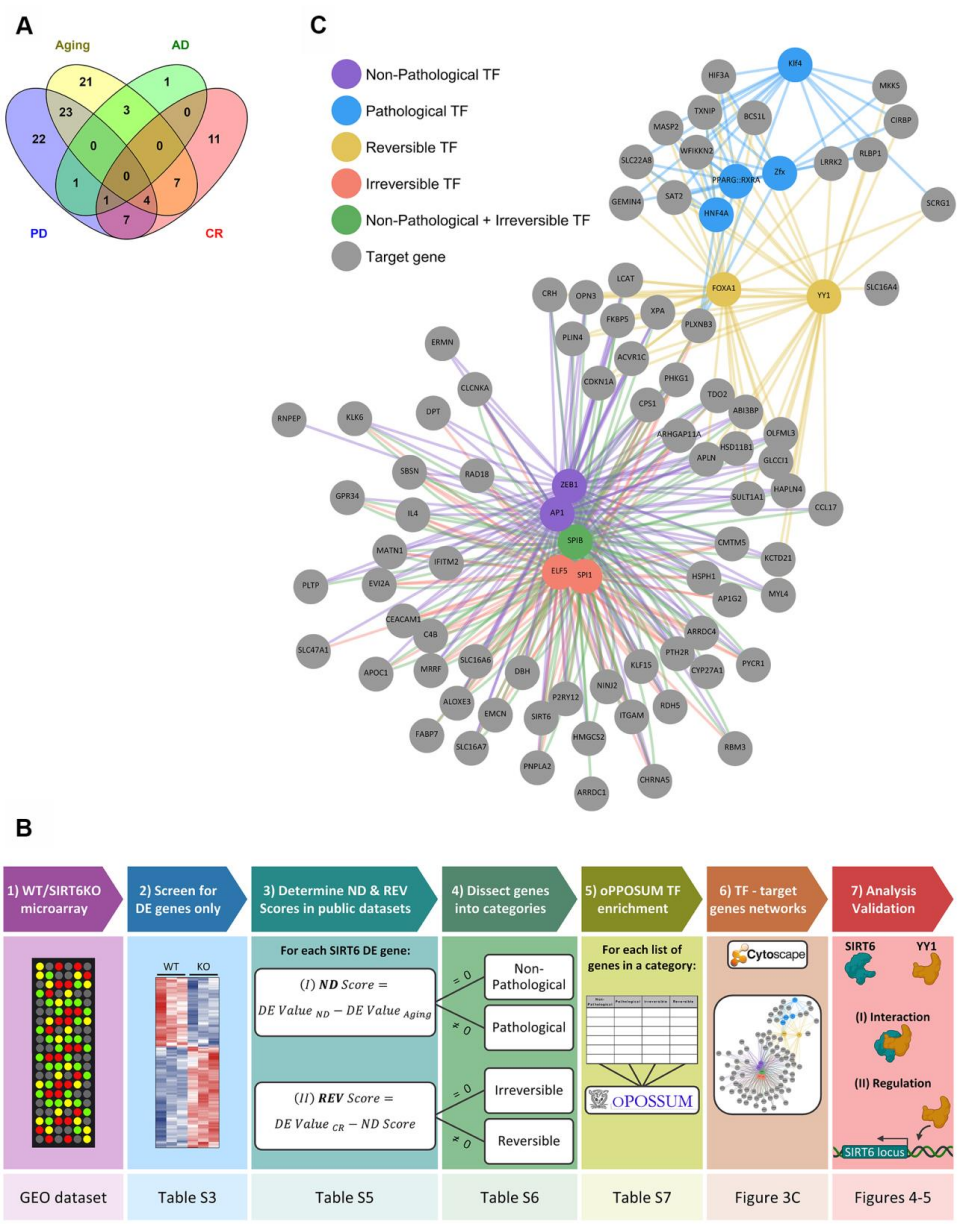
**Selected hubs of brain signature categories.** (**A**) Venn diagram for SIRT6 target genes differentially expressed also in Aging, Alzheimer's disease (AD), Parkinson's disease (PD), or Aged samples under Calorie Restriction (CR) datasets. Numbers represent the number of genes in each section, which change in at least one dataset of a certain group of datasets. (**B**) A scheme of genes analysis pipeline. The lower panels note the Table/Figure in which the results can be found for each step. DE gene – differentially expressed gene; ND and REV Scores – Neurodegeneration and Reversibility Scores; TF – transcription factor. Figure was created with BioRender.com. (**C**) Manually chosen TFs (out of the top TFs per signature category) and their target genes represent the hubs of brain signature categories.

To further study which of the identified genes is associated with either pathological or non-pathological aging, we analyzed all the significantly affected genes in our SIRT6KO mouse brains (only when compared to WT brains; Supplementary Table 3), in publicly available gene expression datasets of normal and pathological brain aging (Supplementary Table 4). Thus, we use SIRT6KO as a focusing lens on particular genes of interest that might be affected with either aging or pathological aging. According to these datasets, affected genes showed a substantial overlap between SIRT6KO and the different conditions (Figure 3A): 58 genes that were differentially expressed in SIRT6KO were also differentially expressed in at least one Aging dataset; 6 genes similarly overlapped with AD datasets and 58 genes overlapped with PD dataset. Since CR is attributed to healthy aging, we used CR as a negative signature for pathological aging (with 30 overlapping genes).

We then dissected our SIRT6KO differentially expressed genes into 4 categories, based on their changes in these public datasets: Non-Pathological, Pathological, Reversible and Irreversible. Each gene was attributed to either Non-Pathological or Pathological category based on its Neurodegeneration Score (ND Score), which represents its expression in neurodegeneration compared with healthy aging. Then, each gene was attributed to either Reversible or Irreversible categories based on its Reversibility Score (REV Score), which represents the change in gene expression caused by caloric restriction. The genes of each category, including their ND and REV Scores can be found in Supplementary Table 5, and the genes in each category (including their Confidence Scores) can be found in Supplementary Table 6. For a simplified scheme of the analysis pipeline, see Figure 3B (for additional details see Methods).

### Non-pathological gene expression changes

Some genes are differentially expressed in similar manners in datasets of both pathological and non-pathological aging cases. This observation suggests that these gene expression changes are merely associated with the patient’s age rather than with the disease. Genes in this category change in a certain direction in both normal and pathological aging.

For example, *C4b* is a gene upregulated with aging in 4 datasets, as well as in 1 AD dataset (ND Score = 0), meaning that C4b is elevated with aging *per se* - an observation that was shown previously [30]. Interestingly, high C4b-binding protein, which inhibits C4b, contributes to the increased disposition to inflammation with age [31], suggesting C4b as a factor whose increase is important in aging and may be relevant to the increased inflammation seen in aging brains.

### Gene expression changes in pathological aging

This category consists of genes that are affected differently by normal or pathological aging. They belong to one the following groups: (1) Genes that change in neurodegeneration in one direction and either do not change or change in the opposite direction in normal aging; or (2) genes that change in aging but do not change in neurodegeneration. Our results are compatible with recent reports of association between some of these genes with aging.

For example, H2-D1 has an ND Score of -1.16, meaning it is upregulated in Aging datasets but downregulated in neurodegeneration. Consistent with our results, the expression of *H2-D1* was previously associated with aging in the human brain and with the upregulation of proinflammatory factors [32].

### Reversible and irreversible gene expression changes in response to CR

The Reversible category includes both Non-Pathological and Pathological genes, whose expression can be reversed using CR. We assume that Reversible Pathological genes may have protective roles against pathological aging. Thus, this list can be of great interest to determine possible targets for different treatments.

On the other hand, the Irreversible category includes the genes whose trend in CR did not change when compared to either Pathological or Non-Pathological aging. Of course, this does not mean that they may not be reversed through other treatments rather than calorie restriction.

### Candidate transcription factors specifically regulate the identified categories

Next, we speculated that genes from the defined categories are co-regulated as a group by certain factors. To address this question, we performed enrichment analysis of transcription factors (TF) binding sites and binding site combinations via oPOSSUM [33]. This analysis helped us to predict whether these genes are co-regulated through a common TF candidate, further supporting the association of the gene groups.

We ranked TFs by their Combined Prominence Scores, which represents the number of target gene hits and Z-score in both humans and mice, indicating whether the TF occurs more frequently than expected (see Methods; Supplementary Table 7). This procedure enabled us to identify TFs of interest associated with almost all the genes in a given category, while filtering noise out (TFs that could be randomly associated). The weights given to Z-score and target gene hits were not fine-tuned, so the Combined Prominence Score was a rough estimate of the significance of TFs, aimed to help an expert to pick specific TFs from the top of the list.

We then created a map, for all 4 categories, of specific TFs together with their targets in the category (Figure 3C). The TFs were chosen manually, based on a particular interest or whether they were unique for one category. Interestingly, some of these TFs were already shown to be linked to aging and neurodegeneration, among them YY1, KLF4, and ELF5 [34–38].

One of the main TFs in the Irreversible category was SPI1. This TF was found to regulate AD-associated genes and replicative senescence – an irreversible process [39, 40]. Most of SPI1 target genes are Non-Pathological. In the Reversible category, FOXA1 was a central regulator. This TF was also shown to be related to cellular neurodegenerative phenotypes and senescence [41–44], and many of its targets are Pathological. Thus, in both categories we can see senescence-associated changes. However, in aging, it seems that these changes are irreversibly directed by SPI1, while in pathology, these changes are regulated by FOXA1 and can be prevented by CR.

### SIRT6 and YY1 form a complex that regulates common processes

**Figure 4:**
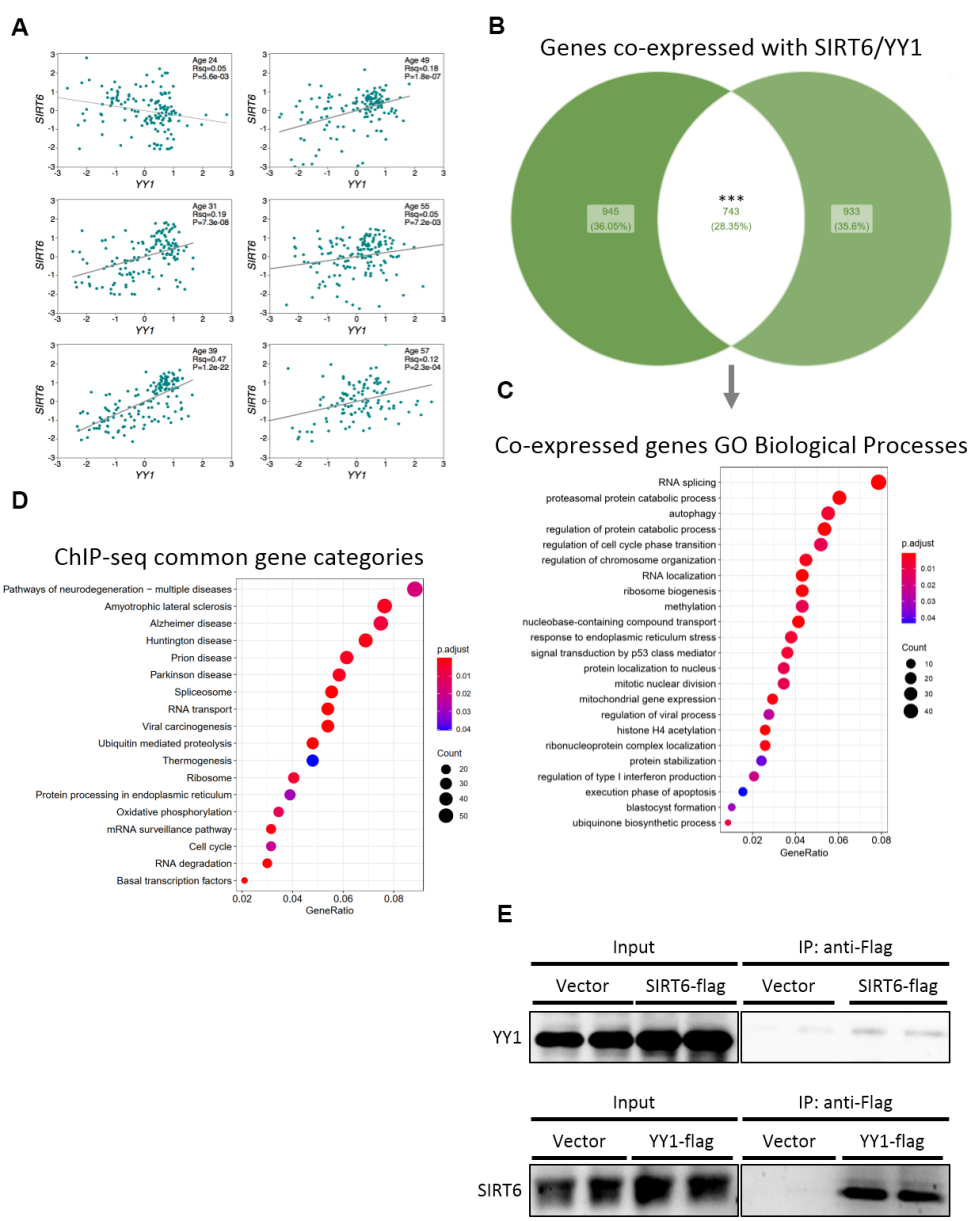
**SIRT6 and YY1 present high similarity in targets.** (**A**) Expression correlations in brains of 6 different donors, based on Allen Brain Atlas microarray data. Each graph is of a different donor and each point on the graph is a different spot of the donor’s brain. (**B**) Venn diagram of the top 2000 genes co-expressed with either SIRT6 or YY1, and their overlapping. (**C**) Enriched GO Biological Processes categories the overlapping genes are part of. (**D**) KEGG enrichment analysis of genes that present peaks in both SIRT6 and YY1 ChIP-seq data (2 cell lines per protein). (**E**) Western blots of co-immunoprecipitation experiments of SIRT6 and YY1. *** - p-value<0.001.

To validate the importance of SIRT6 in our results and in pathological aging, we focused on one irreversible TF – YY1. First, we tested whether SIRT6 and YY1 correlate with similar pathways and cellular functions. We found positive correlations between mRNA levels of these two genes in different brain tissues in 5 out of 6 donors (Figure 4A). Next, we crossed lists of genes that are co-expressed with either SIRT6 or YY1 in the brain (the top 2000 co-expressed genes), based on the assumption that genes that are expressed together are needed for common pathways. From this analysis, we found 742 genes to be co-expressed with both SIRT6 and YY1 (Figure 4B). These genes take part in several biological processes, pointing out the common pathways affected by SIRT6 and YY1 (Figure 4C and Supplementary Figure 2A, 2B). We also crossed published ChIP-seq data of YY1 and SIRT6 in 2 cell lines and found that, yet again, the genes they are localized to take part in very similar pathways (Figure 4D and Supplementary Figure 2C). Interestingly, these common pathways include several neurodegenerative diseases, and even though the cells were not from neuronal origin, they overlap mainly in brain-associated categories. Thus, we show that SIRT6 and YY1 take part in many overlapping processes and functions, many of which are related to pathological brain aging.

Since SIRT6 and YY1 correlate in humans as well as share many common functions and pathways, we speculated that they act together in gene regulation, thus explaining the vast similarities. To assess this, we co-immunoprecipitated YY1 or SIRT6 and found that they bind each other forming a complex (Figure 4E and Supplementary Figure 2D). To conclude, our results shed light on the interaction between SIRT6 and YY1 and on the importance of this interaction in healthy brain aging.

### YY1 regulates SIRT6 expression

**Figure 5:**
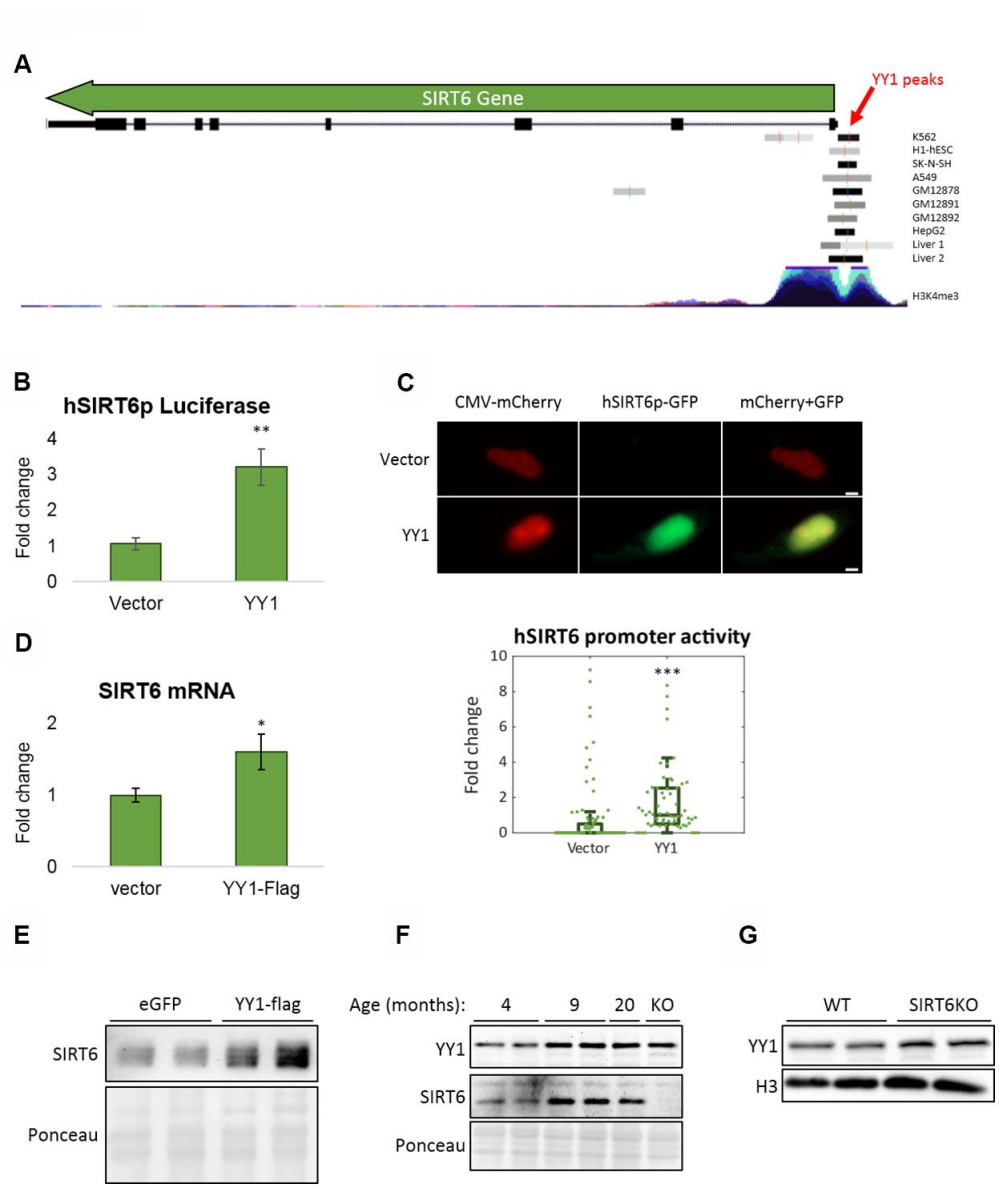
**YY1 regulates SIRT6 promoter.** (**A**) YY1 ChIP-seq data in SIRT6 locus. *SIRT6* locus is marked with a green arrow; YY1 peaks are marked in black or grey shades; tested cell lines are marked on the right side of each YY1 peak. (**B**) hSIRT6p-Firefly Luciferase assay with an empty vector or YY1-Flag overexpression vector. Chosen promoter region – 1000 bases before TSS and 2000 bases after (including the first 2 exons and the intron in between). (**C**) Human SIRT6 promoter activation tested using a hSIRT6p-GFP-CMV-mCherry vector, co-transfected with either empty or YY1-overexpressing vector. Upper panel – representative cell images; lower panel – an unbiased quantification of GFP/mCherry ratio per cell. Box plots represent quartile range, whiskers represent 10% and 90% of datapoints, horizontal line represents median. (**D**) SIRT6 mRNA levels in cells transfected with either an empty or YY1-flag overexpression vector. (**E**) SIRT6 protein levels in cells transfected with either eGFP- or YY1-overexpressing vectors. (**F**) Brains of WT mice at different ages and SIRT6-KO mouse (serves as a control for SIRT6 specificity). (**G**) Protein blots of WT or SIRT6-KO SH-SY5Y cells. * - p-value < 0.05, ** - p-value < 0.01, *** - p-value < 0.001.

To further understand the connection between SIRT6 and YY1, we tested if YY1 regulates SIRT6. First, according to published YY1 ChIP-seq data, YY1 localizes to *SIRT6* promoter (Figure 5A). Importantly, this localization is not common in the region flanking *SIRT6* gene locus, emphasizing the specificity of YY1 to SIRT6 promoter (Supplementary Figure 3A). We then constructed a Firefly Luciferase-based vector controlled by human SIRT6 promoter (hSIRT6p-Fluc) and co-transfected this vector with either empty or YY1-overexpressing vectors. We found a ~3-fold increase in hSIRT6 promoter activity under YY1 overexpression (Figure 5B). However, since YY1 is a very potent transcription factor, it had an adverse effect also on the Renilla Luciferase null-promoter control, preventing us to use Renilla to normalize the Firefly activity. To overcome this issue, we constructed a bicistronic plasmid with human SIRT6 promoter regulation of GFP expression and a constitutively expressed mCherry (Figure 5C). Using this vector, we verified the Luciferase-based SIRT6 promoter activation in YY1 overexpression. The increase in SIRT6 transcription was also demonstrated through RT-qPCR and western blots, which present elevated SIRT6 mRNA (Figure 5D) and protein (Figure 5E) levels upon YY1 overexpression. Although the changes in mRNA and protein levels are small, they are consistent in all the methods we have used and present similar trends. Thus, although YY1 might not be the major regulator of SIRT6, it does regulate its expression, and YY1 overexpression consistently increases SIRT6 levels. Hence, we concluded that YY1 directly activates SIRT6 promoter and regulates its expression, emphasizing the importance of YY1 in healthy aging.

To validate the central role of YY1 in aging, we purified proteins from mouse brains at different ages. Indeed, YY1 levels were increased in aging (Figure 5F). Importantly, SIRT6 levels increased in early adulthood (9-month-old mice) but reduce in old mice (20-month-old). This phenomenon was also recapitulated in the SH-SY5Y neuronal cellular model in which SIRT6 was deleted, thus leading to YY1 upregulation (Figure 5G) – implying a feedback loop in which YY1 regulates SIRT6, and SIRT6 is also critical for the regulation of this central TF. Moreover, while our results show that YY1 overexpression increases SIRT6 levels, this connection is lost in aged mice (as SIRT6 levels decrease and YY1 levels increase), pointing out the pathological nature of aging in the form of reduced SIRT6 levels which are not restored by one of its regulators.

## DISCUSSION

SIRT6 was already revealed as an important factor in the aging brain through several pieces of evidence. First, its levels were shown to decrease in the aging brain [45]. Second, the levels of its cofactor NAD^+^ decrease in human brains, further diminishing SIRT6 catalytic activity [46–48]. Moreover, brain-specific SIRT6 KO mice develop learning impairments, increased DNA damage and cell death, and even the appearance of Hyperphosphorylated and acetylated Tau [10, 21], all characteristics and markers of age-dependent neurodegeneration.

Importantly, AD patients show reduced levels of SIRT6 in their brains together with multiple changes in gene expression, while CR upregulates SIRT6 levels, probably providing some protection [10, 49, 50]. Regulation of gene expression usually weakens with age, causing changes that could lead to disease [51, 52]. SIRT6 is involved in both DNA repair and gene expression regulation (usually transcription repression through its deacetylase activity), and it seems to be one of the driving forces in preventing neurodegeneration.

Most neurodegeneration animal models represent only familial cases of disease with specific point mutations however, these mutations are never seen in sporadic cases. Moreover, these models present intrinsic variability among them. Of course, human samples are even more complicated, with different genetic backgrounds and lifestyles. Hence, finding a critical and common signature to animal models and patients with neurodegenerative diseases has been difficult. Therefore, we decided to use SIRT6-KO mice as a model for sporadic (age-related) neurodegeneration, and to analyze the changes occurring in SIRT6-deficient brains (young) as compared to normal young brains and/or aged brains.

The overlap between changes occurring in SIRT6-deficient young brains (as compared to normal young brains and healthy aging samples) with known datasets demonstrated changes in gene expression in aging and age-related pathologies such as PD and AD, and whether CR could reverse some of these changes in an *in-silico* approach. This approach allowed us to identify target genes that change in aging or pathological aging across datasets, and whether these changes could be reversed by CR. We believe these genes could be further investigated to develop diagnostic tools as well as a better understanding of genes with relevant functions in pathological aging.

Moreover, we point toward specific TFs, such as YY1, which could be master regulators of these signatures. YY1 and SIRT6 are not only involved in similar pathways, but they also form a complex. As a TF that regulates reversible genes, YY1 can serve as a potential target for aging treatments, together with its activity shared with SIRT6. This complex can also provide a mechanism through which YY1 gains, at least partially, its gene repression activity as SIRT6 histone deacetylase activity inhibits proximate gene transcription.

Overall, through the focusing lens of genes that are regulated by SIRT6, our work provides novel targets that are common across datasets in aging and pathological aging, and possible common TFs that regulate them. Our results suggest that SIRT6 absence is a driving force in brain pathology. Our work portrays a roadmap of interesting genes that may be involved in pathological aging in humans. It will be important to further validate the changes in human samples and test whether these changes can be detected in a simple test such as blood samples or free rRNA.

## MATERIALS AND METHODS

### RNA extraction

Total RNA was isolated using RNeasy Mini Kit (74104, Qiagen, CA) according to manufacturer instructions. RNA quality was tested by Agilent Bioanalyzer.

### Affymetrix whole exon array

To estimate gene expression levels, we performed a whole exon array using Affymetrix Mouse Exon 1.0 ST Array with RNA from half hemisphere of brains (no olfactory bulb nor cerebellum) of young mice (3-week-old SIRT6-KO mice and their WT age counterpart), as well as 22-26-month-old WT 129/Sv mice. Jackson laboratory catalog numbers: SIRT6-KO – 006050; WT – 000691.

### Analysis of gene expression data

We performed comparative marker selection in gene expression data using AltAnalyze software [53]. Three SIRT6-KO samples were compared with three WT samples.

Detection cutoffs to detect upregulated or downregulated genes are p-value < 0.01 and minimum fold change of 1.75. The alternative cutoff is p-value < 0.05.

### Geneset enrichment analysis (GSEA) and geneset clustering

For both comparisons, we performed pathway enrichment analysis using GSEA approach [54]. We used GSEA analysis to compare each of the following categories: WT, SIRT6-KO and old WT mice. We then looked at the top 50 enriched Genesets in each comparison (i.e., WT vs SIRT6-KO, SIRT6-KO vs WT, WT vs old WT, etc.) and clustered each Geneset to 1-3 general categories they belong to (i.e., cancer, proteostasis, genomic instability etc.). See Supplementary Table 1.

For each comparison, we then counted the number of enriched Genesets in each category. Categories that had up to two Genesets in all the comparisons were minimized into Others 1 category. Others 2 category includes the following categories: heart, drug resistance, transplantation, viruses. See Supplementary Table 2. A hypergeometric test was then performed using R-function phyper.

We noticed that the cancer category appeared in all the comparisons as the first or second most affected category, implying its over-representation in the analysis. This might occur since cancer is a well-studied field and affects many genes. We, therefore, excluded the cancer category from the figure.

### Published datasets compared to the SIRT6KO mice brains dataset

We analyzed nine datasets of transcriptional profiles for different conditions of brain aging (Supplementary Table 4): human and mouse normal aging [55–59]; human and mouse PD datasets [60, 61]; human and mouse AD datasets [62, 63]; mouse CR dataset [56]. All datasets were downloaded from Gene Expression Omnibus [64].

### Venn diagrams of genesets overlapping with SIRT6 differentially expressed targets

A gene that significantly changed in SIRT6KO mice brains, was considered part of a dataset type (Aging, AD, PD and CR) if it was differentially expressed in at least one dataset of that type. Venn diagrams for these lists of differentially expressed genes were obtained using Venn diagram generator Venny 2.1 [65].

### Differentially expressed genes in the published datasets

Differentially expressed genes were identified in each of the nine datasets using unpaired t-test, with FDR correction of 10% or 50% (used if two or fewer genes passed the 10% threshold, as listed in Supplementary Table 4). Results were compared with lists of upregulated and downregulated genes obtained using our SIRT6KO mice dataset (230 genes, with 259 gene names in total; Supplementary Table 3). Some genes did not have probes in some datasets, and some genes had contradicting probe results in some datasets (mainly due to several orthologs in the conversion from human to mouse genes). Genes that did not appear in at least 50% of the Aging datasets or the Neurodegeneration datasets were excluded from the analysis (see Supplementary Table 4 for Aging and Neurodegeneration datasets).

For the 102 remaining genes, a numerical Differential Expression value (DEVal) was set in each dataset: -1 (the gene decreases in the dataset), +1 (the gene increases in the dataset) or 0 (the gene does not change in the dataset). The mean DEVal was calculated in the Aging and Neurodegeneration datasets, from which a 'Neurodegeneration Score' (ND Score) was calculated by subtracting the mean DEVal of Aging datasets from the mean DEVal of Neurodegeneration datasets. The ND Score represents the change in a given gene, by 'shifting' from normal aging to neurodegenerative aging (a positive ND Score means increase in neurodegeneration, a negative Score means decrease, and 0 means no change). A minimal change of +/-0.4 was agreed to be considered a change.

A gene with an ND Score of 0 (or between the thresholds) means it changes in neurodegeneration and in normal aging in a similar fashion. Thus, this gene belongs to the Non-Pathological gene category. If a gene has an ND Score higher than 0.4 or lower than -0.4, it means it does change in neurodegeneration when compared to healthy aging, thus it belongs to the Pathological gene category.

Both Pathological and Non-Pathological categories were then dissected into Reversible and Irreversible genes by comparing them to the Caloric Restriction (CR) Geneset (only the Old+CR vs. Old groups). A gene is reversible if CR reverses its trend of expression: A Reversible gene presents one trend in neurodegeneration or Aging (e.g., a positive value), but reversed trend or 0 (0 or lower, in the same example) in CR. In a similar manner, an Irreversible gene is a gene whose trend does not change in CR. For each of the pathological genes, a Reversibility Score (REV Score) was calculated as the 'shift' caused by CR by subtracting the ND Score from the CR DEVal. All genes, together with their ND and REV Scores, can be found in Supplementary Table 5.

Each gene in each category has a Confidence Score, which represents how many datasets it appears in and how strong is the change it presents in its category.

Confidence Scores for Non-Pathological genes = ([number of occurrences in Aging and Neurodegeneration datasets] / [total number of datasets]) * ([number of Aging and Neurodegeneration datasets the gene does not change in] / [number of occurrences in Aging and Neurodegeneration datasets]).

Confidence Scores for Pathological genes = ([number of occurrences in Aging and Neurodegeneration datasets] / [total number of datasets]) * [ND Score].

For each of the 4 categories, the genes and their Confidence Scores can be found in Supplementary Table 6.

### Transcription factor (TF) network

For each category defined in the Results section, the list of genes was tested for enrichment of transcription factor binding sites and binding site combinations in mice and human via oPOSSUM [33]. We ran the oPOSSUM Single Site Analysis for both Human and Mouse, using the category gene lists. To sort prominent TFs, we calculated a Hit Score that considers hit and non-hit genes: Hit Score = hits / (hits + non-hits).

We then calculated a Normalized Z-Score for gene i = Z_i_ + ABS(MIN(Z_all_)) to allow a simple ranking of all TFs. We then calculated the TF Prominence Score = Hit score_i_ * Z_i_, and multiplied the Prominence Scores of Human with Mouse to get the Combined Prominence Score. The TFs and their related Scores can be found in Supplementary Table 7.

TFs were then ranked by their Combined Prominence Scores, and TFs from the top of the lists were manually chosen for each category to plot Figure 5B. Specific attention was given to TFs that were unique to one category (out of the top 20 TFs in all categories), or to ones known to be aging-related. Then, the target gene hit file of each chosen TF was manually downloaded from oPPOSUM analysis. These files contain Absolute Scores for each TFBS hit of every hit gene. All Absolute Scores of every gene were averaged, and the Mean Absolute Scores served as the weights of TF-gene interactions in Figure 5B. We then used Cytoscape to plot Figure 5B [66].

### SIRT6-YY1 correlations in human brains

SIRT6 and YY1 RNA expression data were obtained from Allen Brain Atlas (https://human.brain-map.org/) RNA Microarray data [67]. A different correlation graph was created for each of the 6 donors. Each point in each graph represents a specific sample of a specific spot in the donor’s brain.

### Venn diagram of genes co-expressed with SIRT6 and YY1 and enrichment analysis of these genes

The top 2000 probes co-expressed with either SIRT6 or YY1 were downloaded using Allen Brain Atlas (https://human.brain-map.org/) human brain microarray data [67]. Since the Atlas contains many overlapping probes for the genes, the unique gene names were scanned out of all the probes in the separated lists. A unified list of genes co-expressed in both YY1 and SIRT6 lists was generated. A Venn diagram, that represents genes co-expressed with either SIRT6 or YY1 as well as genes co-expressed with both, was generated using ggVennDiagram R package. The p-value was calculated using R function phyper.

The list of genes co-expressed with both SIRT6 and YY1 was analyzed using ClusterProfiler R package (functions: enrichGO, enrichKEGG). For enrichGO, all 743 genes served as input. For enrichKEGG, only 629 out of 743 were recognized.

Next, the GO analysis was simplified using ‘simplify’ function with the following parameters: cutoff = 0.5, by = "p.adjust", select_fun = min. The top unique categories appear in the main Figure 4C.

### SIRT6 and YY1 ChIP-seq common genes enrichment analysis

Published SIRT6 and YY1 ChIP-seq data on H1 and K562 cell lines from the ENCODE database were obtained (ENCSR000BMH, ENCSR000BKD, ENCSR000AUS, ENCSR000AUB) [68–70]. BED-files with broad peaks were used. SIRT6 ChIP-seq data had one replicate per cell line, while YY1 data had two replicates per cell line. Intersections between the replicates were found using Bedtools.

Peaks were then annotated to the closest gene using ChIPseeker R package. Significance of overlapping peaks among chosen ChIP-seq data sets was found using enrichPeakOverlap function from the ChIPseeker R package (p-value ~ 0.001). Genes that overlap among all ChIP-seq datasets were further analyzed for common pathways using ClusterProfiler R package, as previously mentioned (function: enrichKEGG). SIRT6 and YY1 ChIP-seq genes overlapping Venn diagram was generated using ggVennDiagram R package.

### YY1 ChIP-seq analysis in *SIRT6* gene locus

YY1 ChIP-seq data was viewed in UCSC Genome Browser [71], using 'Transcription Factor ChIP-seq Peaks' Track. Out of all the TF tracks, only those of YY1 with peak in SIRT6 are presented (9 out of 10 tracks). Additionally, the 'H3K4Me3 Mark' layered track was added to mark SIRT6 promoter region.

### Cell culture

All cells were grown in DMEM (41965039, Thermo-Fisher Gibco®, MA), supplemented with 1% L-glutamine (25030024, Thermo-Fisher Gibco®, MA), 1% Penicillin/Streptomycin antibiotics mix (15140122, Thermo-Fisher Gibco®, MA) and 10% FBS (12657-029, Thermo-Fisher Gibco®, MA). Incubation was in 37° C, 5% CO2.

### Transfections

Transfections were conducted using PolyJet™ *In Vitro* DNA Transfection Reagent (SL100688, SignaGen® Laboratories, MD), according to manufacturer’s protocol.

### SIRT6 and YY1 co-immunoprecipitations

70% confluent SH-SY5Y cultures were transfected with pCMV-Sirt6-Flag and pCMV-YY1-Flag plasmids, mock cells were used as negative controls. 24 hours after transfection, cells were collected and re-suspended in 150mM lysis buffer (150mM KCl, 0.1% Triton X-100, 25mM Tris pH 7.5, 5% glycerol, 0.2mM EDTA, 1mM DTT, 200uM PMSF). Cell lysates were cleared by centrifugation at maximum speed, and the supernatant was incubated with Anti-Flag Affinity gel (A2220, Merck KGaA, Germany), during 2 hours at 4° C under rotatory agitation. After Anti-Flag Affinity gel incubation, the samples were washed 3 times with 150mM lysis buffer, and two times with 300mM Lysis buffer (same as 150mM buffer but with 300mM KCl). Flag-purified proteins were eluted with an excess of flag peptide. Same volumes of eluted samples and input samples were analyzed by immunoblotting.

### Human SIRT6-promoter luciferase assay

Cells were plated and transfected 24 hours afterward with null-T7-RenillaLuc, hSIRT6p-FireflyLuc and either an empty or pCMV-YY1-Flag plasmids. Lysis, luciferase substrates and luminescence readings were conducted using Dual- Luciferase® Reporter Assay System (E1960, Promega Corporation, WI), according to manufacturer’s protocol.

### Human SIRT6-promoter fluorescence assay

Cells were plated in 8-microchmaber slides (10k cells/chamber) and transfected 24 hours afterwards with hSIRT6p-GFP-CMV-mCherry plasmid, together with either an empty or pCMV-YY1-Flag plasmids. One chamber served as a fluorescence control (containing only the fluorescent reporter plasmid) and another chamber served as a background and autofluorescence control (non-transfected cells). They were then fixated in 2% paraformaldehyde 72h post transfections (when fluorescence in both channels was clearly visible in the control chamber). The slide was then visualized and photos were captured using Zeiss fluorescent system (verifying no background signal is visible using the non-transfected chamber). Analysis was conducted using a manually adjusted automated pipeline in CellProfiler program [72], which included only mCherry-positive cells. Once GFP and mCherry signals were obtained, their ratio was calculated and statistical significance was calculated using Mann-Whitney U test (U=1254, Z-score=-6.07, p-value<0.001).

### RNA purification and cDNA reverse transcription

Cells were plated in 6-multiwell plate (200k cells/well) and transfected 24 hours afterwards with either an empty or pCMV-YY1-Flag plasmids. RNA was purified using EZ-RNA II Kit (20-410-100, Biological Industries Israel, Beit Hamek Ltd., Israel), according to manufacturer’s protocol.

Then, cDNA was reverse-transcribed using qScript® cDNA Synthesis Kit (95047, QIAGEN Beverly Inc., MA).

### Quantitative PCR

cDNA levels were measured using LightCycler® 480 Probes Master (04887301001, Roche Molecular Systems, Inc., Switzerland). All probes in use were manufactured by IDT (Integrated DNA Technologies, Inc., IA): human ACTB (Hs.PT.39a.22214847); human SIRT6 (Hs.PT.58.39904591, Hs.PT.58.38621021.g).

### Protein antibodies

anti-SIRT6 and anti-YY1 were diluted 1:1000 in 5% BSA in TTBS, with 0.02% sodium azide. Antibodies that were in use: anti-SIRT6 (12486S, Cell Signaling Technology, Inc, MA), anti-YY1 (ab109237, Abcam plc., UK), anti-H3 (ab1791, Abcam plc., UK).

## Supplementary Material

Supplementary Figures

Supplementary Table 1

Supplementary Table 2

Supplementary Table 3

Supplementary Table 4

Supplementary Table 5

Supplementary Table 6

Supplementary Table 7
