# Supplementary material for: Aging and pathological aging signatures of the brain: through the focusing lens of SIRT6
Source: Aging (Albany NY). 2021 Mar 9;13(5):6420–41. doi: 10.18632/aging.202755 (PMC7993737; doi:10.18632/aging.202755)
Supplement: Supplementary Figures [file aging-13-202755-s001.pdf]

## SUPPLEMENTARY FIGURES

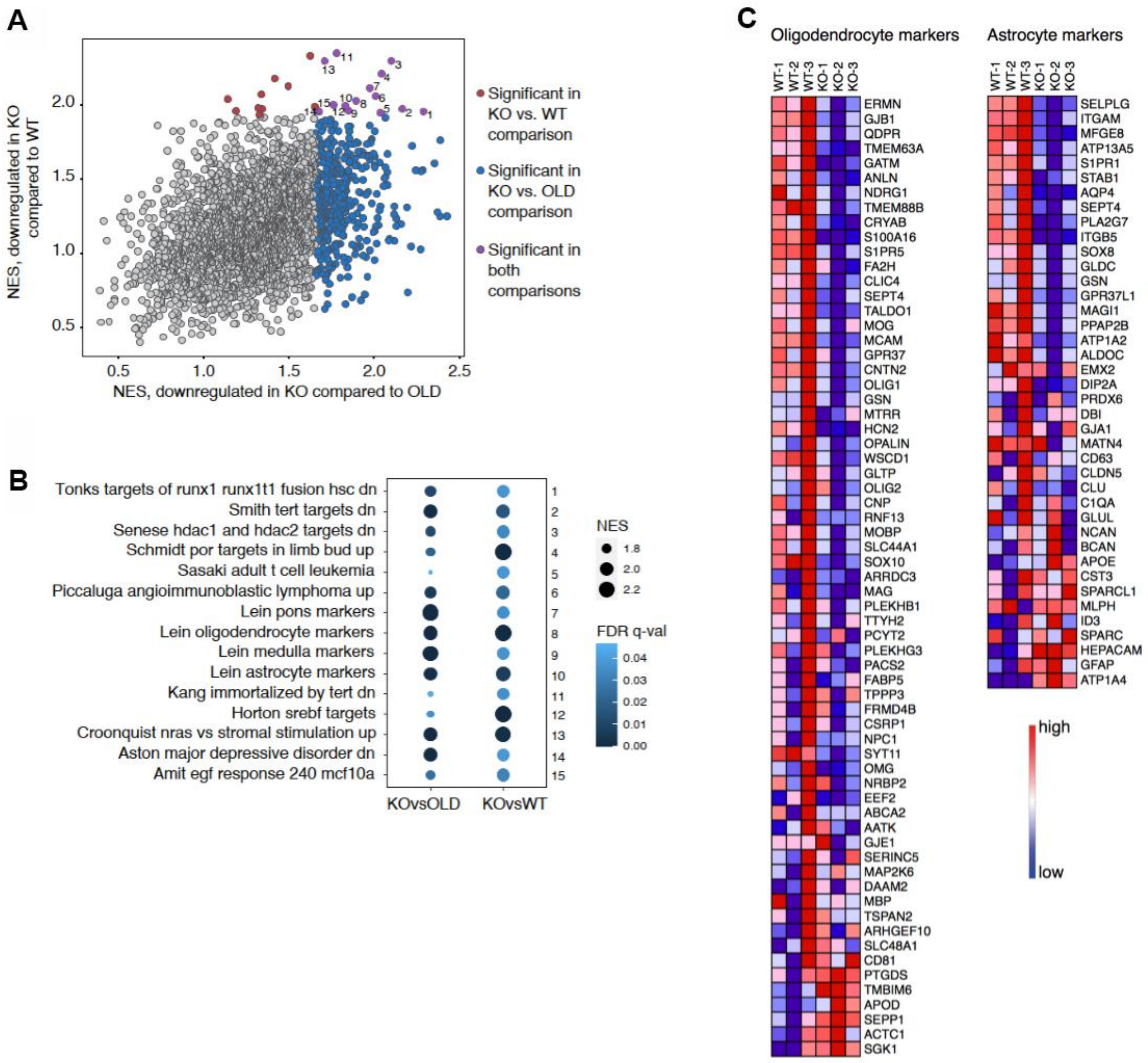

**Supplementary Figure 1. SIRT6 deletion affects several unique gene categories.** (**A**) Normalized Enrichment Scores (NES) calculated in Gene Set Enrichment Analysis (GSEA) for genes downregulated in SIRT6-KO compared to young WT mice (Y-axis) or to old WT mice (X-axis). Dots represent functional categories. Blue dots show significantly downregulated categories in a comparison between SIRT6-KO and old WT mice, while red dots correspond to a comparison between SIRT6-KO and young WT mice. Violet dots highlight categories significant in both comparisons. Significance cutoff: FDR < 0.05. (**B**) Significant categories in both comparisons. Colors represent FDR. Circle sizes are proportional to the NES. (**C**) Expression levels of genes in two significant categories: oligodendrocyte markers (left panel) and astrocyte markers (right panel). Red color corresponds to increased expression, while blue color corresponds to decreased expression.

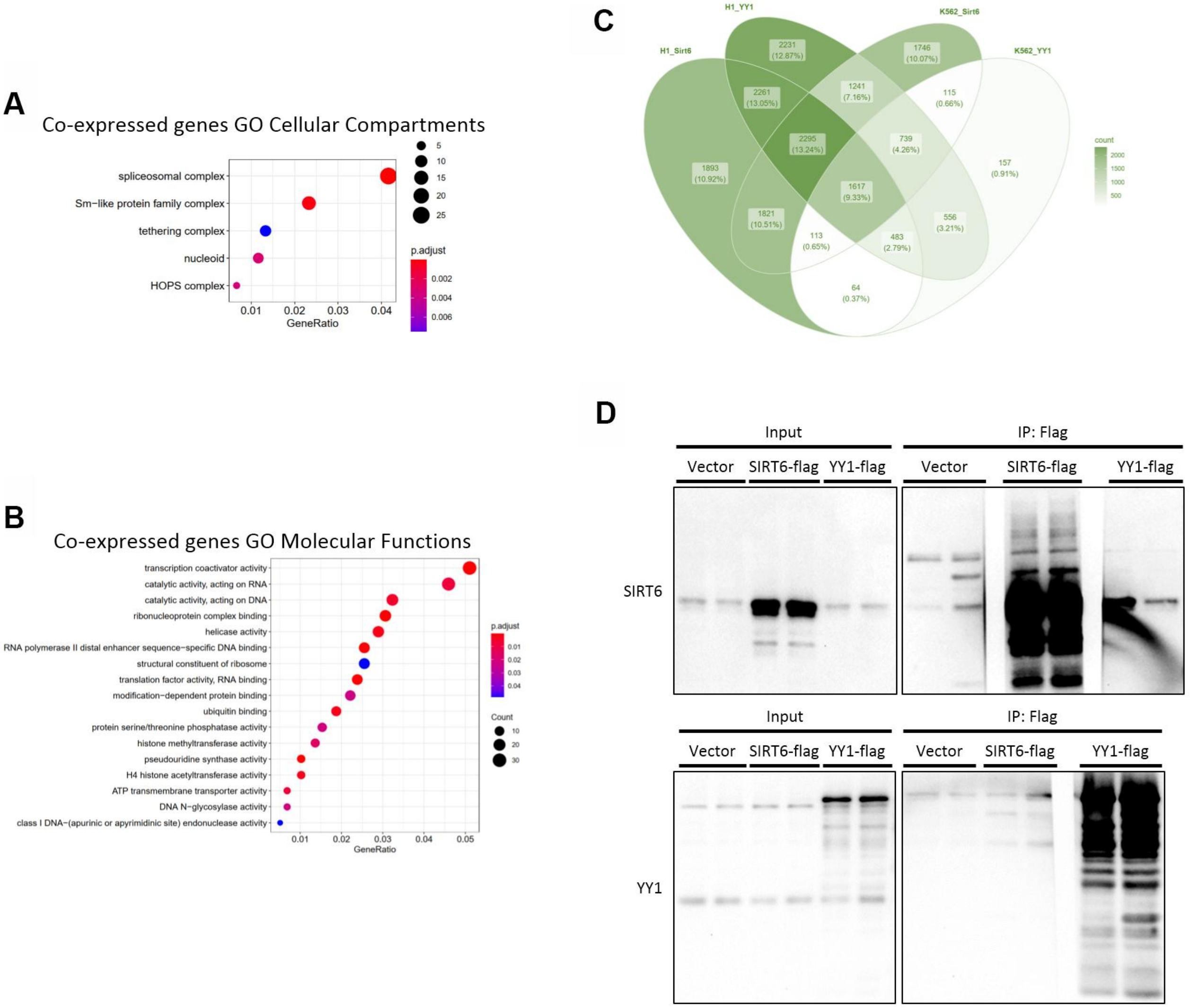


**Supplementary Figure 2. SIRT6 and YY1 present high similarity in targets.** (**A**) Enriched GO Cellular Compartments categories the overlapping genes in Figure 4B are part of. (**B**) Enriched GO Molecular Functions categories the overlapping genes in figure 4B are part of. (**C**) A Venn diagram of SIRT6 and YY1 ChIP-seq genes, overlapping between the 4 used ChIP-seq datasets. Only genes that are shared among the 4 datasets were used in Figure 4D. (**D**) Western blots of SIRT6 and YY1 co-IP showing the transfection and IP efficiency.

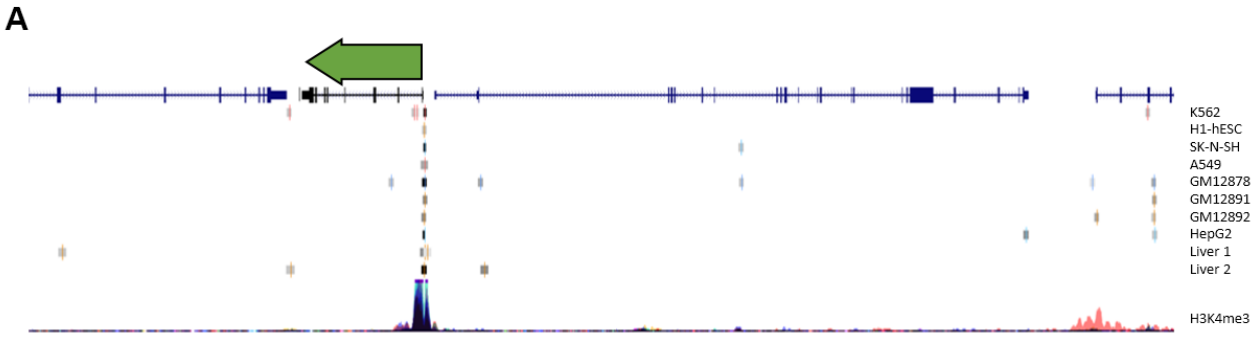


**Supplementary Figure 3. YY1 regulates SIRT6 promoter.** (**A**) YY1 ChIP-seq data in a large region that flanks SIRT6 gene. *SIRT6* locus is marked with a green arrow; YY1 peaks are marked in black or grey shades; tested cell lines are marked on the right side of the panel.
