# Supplementary material for: Aging and pathological aging signatures of the brain: through the focusing lens of SIRT6
Source: Aging (Albany NY). 2021 Mar 9;13(5):6420–41. doi: 10.18632/aging.202755 (PMC7993737; doi:10.18632/aging.202755)
Supplement: Supplementary Table 2 [file aging-13-202755-s003.pdf]

Supplementary Table 2. GSEA clusters summarizing table.

**comparison name**	**WT vs KO**	**WT vs old**	**Old vs KO**	**KO vs WT**	**old vs WT**	**KO vs Old**
**direction**	**downregulated**	**downregulated**	**downregulated**	**upregulated**	**upregulated**	**upregulated**
**up/down in**	**KO**	**Old**	**KO (comp. to Old)**	**KO**	**Old**	**KO (comp. to Old)**
immune system	3	3	20	2	19	0
protein translation	1	0	2	5	7	0
proteostasis	1	1	0	0	0	0
RNA metabolism	1	0	0	2	3	0
lipid metabolism	4	0	1	0	2	1
aging	0	2	4	0	3	0
cell cycle	3	11	6	21	2	12
differentiation	2	3	1	2	1	5
brain region	4	0	7	0	4	0
brain development	3	5	0	0	0	3
genomic instability	0	1	0	3	0	5
DNA synthesis	0	0	0	8	0	0
epigenetic modifications	4	2	1	0	0	5
development	2	3	0	0	0	0
learning	3	4	0	0	0	2
neuronal system	6	2	0	0	0	2
cell-cell interaction	6	5	0	1	0	5
**cancer**	9	26	17	13	17	19
**Others 1**	**9**	**8**	**6**	**6**	**5**	**9**
**Others 2**	0	1	5	2	9	5
