# Supplementary material for: Aging and pathological aging signatures of the brain: through the focusing lens of SIRT6
Source: Aging (Albany NY). 2021 Mar 9;13(5):6420–41. doi: 10.18632/aging.202755 (PMC7993737; doi:10.18632/aging.202755)
Supplement: Supplementary Table 4 [file aging-13-202755-s005.pdf]

Supplementary Table 4. Public datasets information.

Dataset	Condition	Organism	Compared samples	FDR threshold
GDS707	Aging	human	Old (12) VS Young (9)	0.5
GSE5078	Aging	mouse	Old (14) VS Young (9)	0.1
GSE45044	Aging	mouse	Old (4) VS Young (4)	0.1
GSE68169	Aging	mouse	Old (3) VS Young (3)	0.1
GSE11291	Aging	mouse	Old (5) VS Young (5)	0.1
GSE7621	PD	human	PD_patients (16) VS Control_patients (9)	0.5
GSE60413	PD	mouse	PD_model (3) VS WT_model (3)	0.1
GSE28146	AD	human	AD_patients (15) VS Control_patients (8)	0.5
GSE50521	AD	mouse	AD_model (3) VS WT_model (3)	0.5
GSE11291	Calorie Restriction	mouse	OldCR (5) VS Old (5)	0.1
